# In vivo and in silico characterization of apocynin in reducing organ oxidative stress: A pharmacokinetic and pharmacodynamic study

**DOI:** 10.1002/prp2.635

**Published:** 2020-08-05

**Authors:** Fangfei Liu, Lampson M. Fan, Nicholas Michael, Jian‐Mei Li

**Affiliations:** ^1^ School of Biological Sciences University of Reading Reading UK; ^2^ The Royal Wolverhampton NHS Trust Wolverhampton UK; ^3^ Chemical Analysis Facility University of Reading Reading UK

**Keywords:** apocynin, in vivo, NADPH oxidase, obesity, PKPD, reactive oxygen species

## Abstract

Apocynin has been widely used in vivo as a Nox2‐contaninig nicotinamide adenine dinucleotide phosphate oxidase inhibitor. However, its time‐dependent tissue distribution and inhibition on organ reactive oxygen species (ROS) production remained unclear. In this study, we examined apocynin pharmacokinetics and pharmacodynamics (PKPD) after intravenous (iv) injection (bolus, 5 mg/kg) of mice (CD1, 12‐week). Apocynin was detected using a HPLC coupled to a linear ion‐trap tandem mass spectrometer. Apocynin peak concentrations were detected in plasma at 1 minute (5494 ± 400 ng/mL) (*t*_1/2_ = 0.05 hours, clearance = 7.76 L/h/kg), in urine at 15 minutes (14 942 ± 5977 ng/mL), in liver at 5 minutes (2853 ± 35 ng/g), in heart at 5 minutes (3161 ± 309 ng/g) and in brain at 1 minute (4603 ± 208 ng/g) after iv injection. These were accompanied with reduction of ROS production in the liver, heart and brain homogenates. Diapocynin was not detected in these samples. Therapeutic effect of apocynin was examined using a mouse model (C57BL/6J) of high‐fat diet (HFD, 16 weeks)‐induced obesity and accelerated aging. Apocynin (5 mmol/L) was supplied in drinking water during the HFD period and was detected at the end of treatment in the brain (5369 ± 1612 ng/g), liver (4818 ± 1340 ng/g) and heart (1795 ± 1487 ng/g) along with significant reductions of ROS production in these organs. In conclusion, apocynin PKPD is characterized by a short half‐life, rapid clearance, good distribution and inhibition of ROS production in major organs. Diapocynin is not a metabolite of apocynin in vivo. Apocynin crosses easily the blood‐brain barrier and reduces brain oxidative stress associated with metabolic disorders and aging.

**Abbreviations**
AIC: Akaike's information criterion
AIC_c_: AIC corrected
BIC: Bayesian information criterion
CNS: central nerve system
DHE: dihydroethidium
HFD: high‐fat diet
HPLC‐LTQ‐MS/MS: high‐performance liquid chromatography coupled to a quadrupole‐linear ion‐trap tandem mass spectrometry
iv: intravenous
MDA: malondialdehyde
NADPH: nicotinamide adenine dinucleotide phosphate
NCD: normal‐chow diet
Nox2: Nox2‐containing NADPH oxidase
PKPD: pharmacokinetics and pharmacodynamics
ROS: reactive oxygen species
SOD: superoxide dismutase

## 1 INTRODUCTION

Multiple organ oxidative stress attributable to increased reactive oxygen species (ROS) production plays an important role in the development of age‐related metabolic, cardiovascular, and neurodegenerative diseases.^1^, ^2^ Although there are several enzymatic sources of ROS production in organs, a Nox2‐containing nicotinamide adenine dinucleotide phosphate (NADPH) oxidase (Nox2) has been found to be an important source of increased ROS production in these disease conditions.^1^, ^3^, ^4^, ^5^ Under physiological conditions, Nox2 is not activated and the low levels of ROS generated are mainly used for cellular signaling. However, Nox2 is activated under pathological conditions such as inflammation, metabolic disorders, ischemic reperfusion injury, and aging.^1^, ^4^ Activated Nox2 generates large amount of $O_{2}^{\cdot ‐}$ causing oxidative damage to tissues and organs. Knockout Nox2 protects cerebral vasculature and neurons from oxidative damage and preserved locomotor function in aging mice.^5^

Apocynin (4‐hydroxy‐3‐methoxyacetophenone) is a naturally occurring compound isolated from the roots of Picrorhiza kurroa Royle ex Benth.^6^, ^7^ It has a molecular weight of 166.17 Da, a pKa value of 8.17 and a log *P* value (partitioning coefficient in n‐octanol/water) of 0.83, which give apocynin easy access to the cell membrane and its target sites.^7^ Apocynin acts by blocking p47^phox^ (a major regulatory subunit of Nox2) forming a complex with cytochrome b, which inhibits $O_{2}^{\cdot ‐}$ production by Nox2 NADPH oxidase.^6^ Apocynin had been widely used as a Nox2 inhibitor in animal models of oxidative stress‐related cardiovascular, metabolic, liver and neurodegenerative diseases.^8^, ^9^, ^10^, ^11^ However, there are discrepancies in the literature regarding the action of apocynin to reduce oxidative stress. For example, several studies had reported that diapocynin was a metabolite of apocynin, that apocynin acted through diapocynin.^12^, ^13^ Others claimed that apocynin was an antioxidant rather than a Nox2 inhibitor in the vascular system since diapocynin was not formed in vascular endothelial cells or smooth muscle cells.^14^ One study even found apocynin to promote ROS production in mouse embryonic cells.^15^ There has been a lack of pharmacokinetics and pharmacodynamics (PKPD) characterization of apocynin as a Nox2 inhibitor to inhibit ROS production in major organs. No study had examined the ability of apocynin to cross blood‐brain barrier (BBB) to reduce brain oxidative stress in disease conditions. Therefore, the objectives of the study were to establish in vivo the PK profile of apocynin in plasma, in urine and in major organs after iv injection and the PD profile of apocynin to inhibit $O_{2}^{\cdot ‐}$ production in these organs.

In this study, we investigated the PKPD of apocynin in mice, which are the mostly used animals in experimental medicine. Time‐dependent tissue distribution of apocynin in the plasma, urine, liver, heart, and brain was examined after intravenous (iv) injection and detected using a high‐performance liquid chromatography (HPLC) coupled to a linear ion‐trap tandem mass spectrometer (HPLC‐MS/MS). PK simulation and modeling were performed using Phoenix WinNonlin 8.1 program. We evaluated the time‐dependent inhibitory effects of apocynin on ROS production by liver, heart, and brain tissues after iv injection. The therapeutic potential of apocynin in reducing major organ (heart, liver, and brain) oxidative stress was further examined using a mouse model of high fat‐diet (HFD) induced obesity and accelerated aging.^16^ The novel information provided by the current study helps the future application of apocynin or its derivatives as potential drugs to treat oxidative‐stress‐related diseases.

## 2 MATERIALS AND METHODS

### 2.1 Chemicals and Reagents

Apocynin (purity ≥ 98%), diapocynin, and phenacetin (N‐(4‐ethoxyphenyl) acetamide) (used as an internal stand for detection using HPLC‐MS/MS) were obtained from Sigma Aldrich (UK). Methanol, water, acetonitrile of LC‐MS grade, and ethanol of HPLC grade were purchased from Thermo Fisher Scientific, UK. Dihydroethidium (DHE) was from Invitrogen (UK). Antibodies against Nox2 were from Santa Cruz Biotechnology (UK). All other reagents were purchased from Sigma‐Aldrich unless specified in the text.

### 2.2 In vivo PKPD experiments and mouse model of high‐fat diet‐induced metabolic disorders

All studies were performed in accordance with the protocols approved by the Home Office under the Animals (Scientific Procedures) Act 1986, UK. For the PKPD study, male CD1 mice (Charles River Ltd) at 12 weeks of age with a body weight range between 25 and 30 g were used for the study. Mice were fasted 12 hours before bolus iv injection of apocynin at 5 mg/kg body weight (dosing volume 5 mL/kg) via a lateral tail vein. Control mice were injected with vehicle (solvent of apocynin) at each time points. Four mice/per time points were sacrificed at 0, 1, 2.5, 5, 15, 30, 60 minutes and then at 3, 6, 12, and 24 hours after iv injection of apocynin. Blood samples were collected and centrifuged at 2000 *g*, 4°C for 10 minutes for plasma collection. Urine samples were collected from bladders. Tissues were harvested for further experiments.

The mouse model of high‐fat diet (HFD‐induced metabolic disorders and accelerated aging was established as described previously.^8^ Littermates of WT and Nox2KO mice (Jackson Laboratory) on a C57BL/6J background at 7 months of age were randomly assigned (n = 6/per group) to a HFD containing 45% kcal fat (Special Diets Services), or to a normal chow diet (NCD) containing 9.3% kcal fat (LabDiet Ltd) for 16 weeks. Apocynin was supplied in drinking water (5 mmol/L) for the treatment group. Body weights were measured weekly. Mice were sacrificed at 11 months of age and organs were harvested for further experiments.

### 2.3 HPLC‐MS/MS detection of apocynin, method validation, and sample extraction

The detection of apocynin in tissue sample was performed as described previously with some modifications.^17^ In brief, apocynin was detected using an Accela HPLC coupled to a linear ion‐trap tandem mass spectrometry (LTQ‐Orbitrap XL; Thermo‐Fisher Scientific) (HPLC‐MS/MS) in the negative ion mode. Phenacetin (500 ng/mL) was used as an internal standard in every vial. A reversed‐phase 50 × 2.1 mm ID, 1.9 mm particle, 175 Å pore C18 Hypersil Gold column (Thermo Scientific) was used with injection volume set at 5 µL for the separation of apocynin and phenacetin. Calibration curve used for PK analysis was constructed by spiking apocynin in mouse plasma using eight calibrators ranging from 1 to 10000 ng/mL with great reproducibility and *R* ^2^ ≥ 0.996 for subsequent 5 separate experiments on different days. Best fit curve was achieved using linear regression model (1/*ꭓ* ^2^ weighting factor) which showed minimum percentage relative error. Limit of detection for apocynin was set up at signal to noise ratio ≥ 3,^18^ which is 1 ng/mL in our system. The limitation of quantification was set up at signal to noise ratio ≥ 10, which is 10 ng/mL in our system. Orbitrap component was used for the full scan spectra of apocynin and diapocynin.

### 2.4 PK simulation and model analysis using Phoenix WinNonlin 8.1

PK parameters simulation was performed using the Phoenix WinNonlin 8.1 software (Certara.com). The values of plasma apocynin concentrations vs actual sampling time (1‐30 minutes) after iv injection were modeled by non‐compartmental analysis.^19^, ^20^ The calculated parameters included peak concentration (*C*_max_); AUC_1‐30 min_ and AUC_0‐∞_ (time 0 to infinity); elimination rate constant (kel), t_1/2_, and plasma clearance (CL). Data collected from sampling time 1‐360 minutes after iv injection were then pooled (n = 3) and tested against various compartmental models for a best fit using Phoenix WinNonlin 8.1 software. Parameters included the Akaike's information criterion (AIC), Bayesian information criteria (BIC) and corrected AIC (AIC_c_) calculated using the equation: AIC_c_ = AIC +2 × nParm (nParm + 1)/(nObs − nParm − 1).^21^

### 2.5 LogBB calculation

LogBB of apocynin was calculated as described previously^22^ using the equation: LogBB = Log (C_brain_/C_plasma_). We used the values from 1 to 30 minutes detected in both plasma and brain tissues for LogBB calculation since apocynin plasma concentration was fallen to ~10 ng/mL (limit of quantification) after 30 minutes of iv injection, n = 3 mice/per time point.

### 2.6 Measuring ROS production

ROS production was measured using tissue homogenates by three complementary techniques: lucigenin (5 µmol/L)‐chemiluminescence (Molecular Devices); DHE (2 µmol/L) fluorescence on brain tissue sections, and brain tissue lipid peroxidation by malondialdehyde (MDA) assay as described previously.^5^ The specificity of detection of $O_{2}^{\cdot ‐}$ was confirmed by adding tiron (10 mmol/L), a non‐enzymatic $O_{2}^{\cdot ‐}$ scavenger. The enzymatic sources of $O_{2}^{\cdot ‐}$ production were identified using inhibitors targeting nitric oxide synthase (L‐N^G^‐Nitro arginine methyl ester, L‐NAME, 100 μmol/L), the mitochondrial complex‐1 enzymes (rotenone, 50 μmol/L), xanthine oxidase (oxypurinol, 250 μmol/L), flavo‐proteins (diphenyleneiodonium, DPI, 20 μmol/L), or superoxide dismutase (SOD) (200 U/mL). The DHE fluorescence was captured using a Nikon fluorescence microscope (Eclipse Ti2‐E). The fluorescence intensity was quantified from at least three random images per section with three sections/per sample.

### 2.7 Mouse model of HFD‐induced metabolic disorder and aging

The HFD study was performed exactly as described previously.^8^ Littermates of WT and Nox2KO mice on a C57BL/6J background (Jackson Laboratory, USA) were bred in our institution from heterozygotes and genotyped. Male mice at 7 months of age were randomly assigned (n = 6/per group) to a HFD: 45% kcal fat, 20% kcal protein and 35% kcal carbohydrate (Special Diets Services), or a NCD: 9.3% kcal fat, 25.9% kcal protein, and 64.8% kcal carbohydrate (LabDiet Ltd) for 16 weeks. Apocynin was supplied in drinking water (5 mmol/L). Body weights were measured weekly. Mice (at 11 months) were fasted 8 hours before being sacrificed by an overdose of pentobarbital. The body weights were recorded and organs were harvested.

### 2.8 Immunofluorescence microscopy

The experiments were performed exactly as described previously.^23^ Midbrain (containing hippocampus and ventral tegmental area regions) cryosections were used for the experiments. Primary antibodies were used at 1:250 dilution. Bovine serum albumin (2%) was used in the place of primary antibodies as a negative control. Biotin‐conjugated secondary antibody was used at 1:1000 dilution. Specific binding of antibodies was detected by streptavidin‐Cy3. Images were captured using a Nikon fluorescence microscope (Eclipse Ti2‐E). Fluorescence intensities were quantified as described above.

### 2.9 Data analysis

For the in vivo PKPD, three mice were used for each time point and a total of 8 time points were used at 0, 1, 2.5, 5, 15, 30, 60, and 180 minutes after iv injection of apocynin. Data were expressed as the means ± SD unless otherwise specified in the figure legends. For the HFD study, six mice were used for each group. Statistical analysis was performed using one‐way ANOVA, followed by Bonferroni *post hoc* tests. *P* < .05 was considered statistically significant.

## 3 RESULTS

### 3.1 Detection of apocynin and diapocynin in tissues after IV bolus injection

**FIGURE 1:**
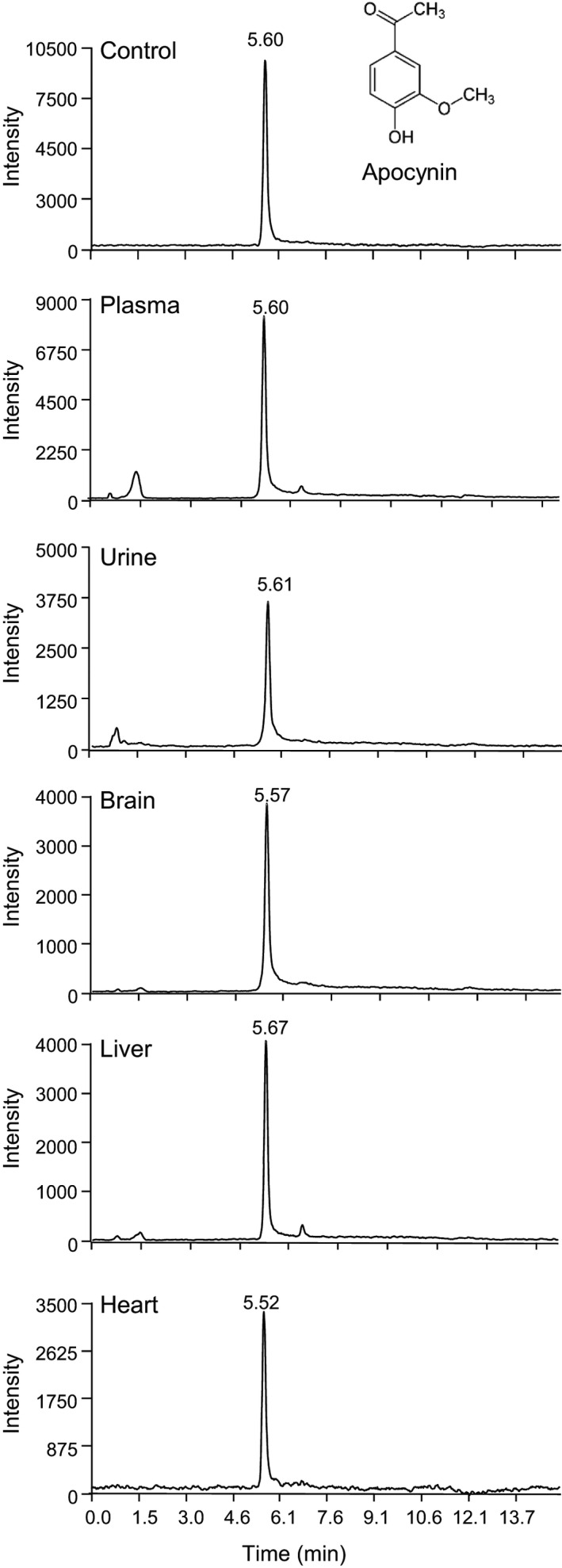
Representative chromatograms of apocynin detected using HPLC‐MS/MS. Control sample (top panel) was apocynin (500 ng/mL) spiked into mouse plasma and was detected as a prominent peak at ~5.5‐5.7 min. Apocynin was detected in the plasma, brain, liver, and heart samples harvested at 5 min and in urine samples harvested at 15 min after intravenous injection. HPLC‐MS/MS, HPLC coupled to a quadrupole‐linear ion‐trap tandem mass spectrometry

Full scan of apocynin and diapocynin were performed using LTQ Orbitrap mass spectrometry under the negative heated electrospray ionisation mode. Apocynin was detected as a prominent peak at m/z 165.0543 and diapocynin was detected as a prominent peak at m/z 329.1058 (Figure S1). Apocynin (500 ng/mL) spiked in mouse plasma was then used as control for the detection using HPLC‐MS/MS, which showed a prominent single symmetrical peak appeared at a retention time of approximately 5.5‐5.7 minutes. Apocynin was detected in the plasma, urine, and the tissue homogenates of the brain, liver, and heart after iv bolus injection (Figure 1).

**FIGURE 2:**
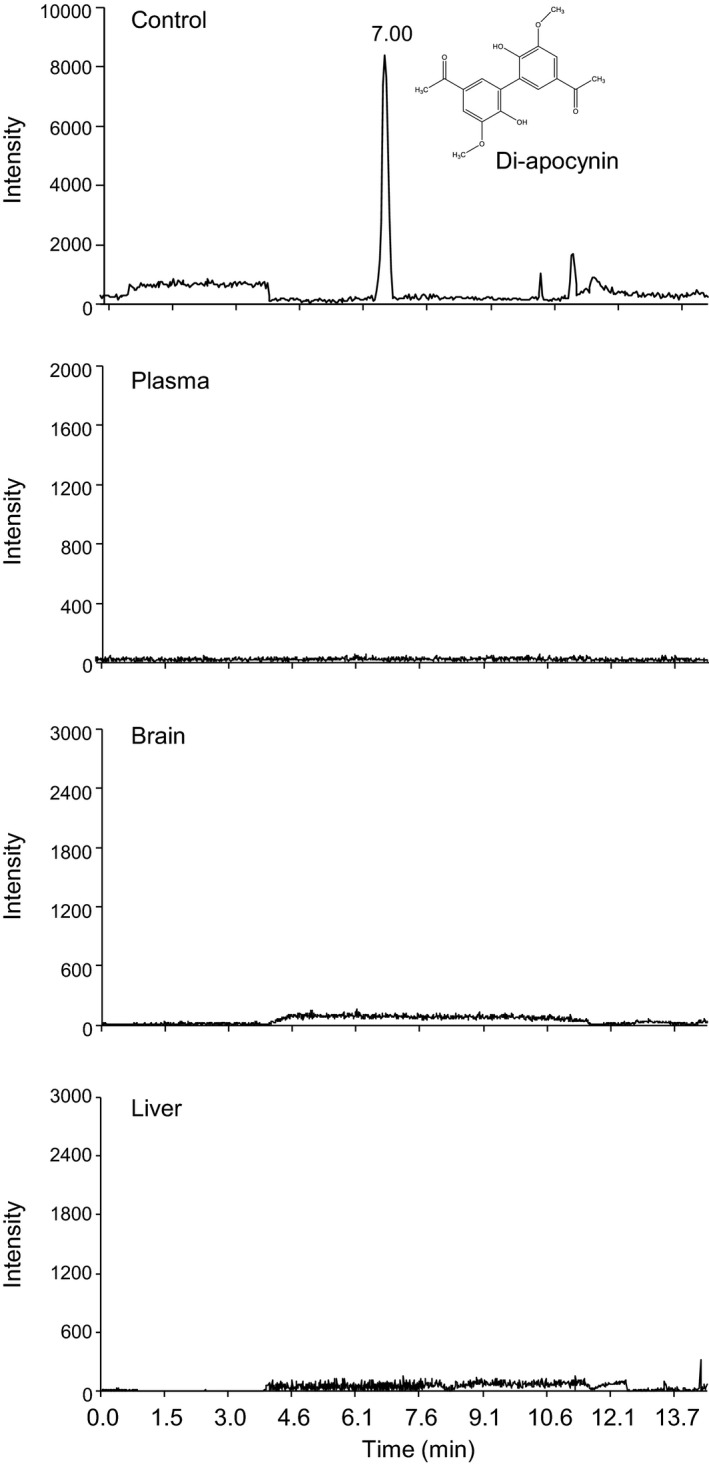
Representative chromatograms of diapocynin detected using HPLC‐MS/MS. Control sample (top panel) was diapocynin (500 ng/mL) spiked into mouse plasma and was detected as a prominent peak at ~6.8‐7.2 min. Diapocynin was not detected in the plasma, brain, and liver samples after intravenous injection of apocynin. HPLC‐MS/MS, HPLC coupled to a quadrupole‐linear ion‐trap tandem mass spectrometry

Apocynin had been reported to form diapocynin, in the presence of H_2_O_2_ and myeloperoxidase, in order to be active.^14^ Therefore, we examined the formation of diapocynin in the samples of apocynin using HPLC‐MS/MS (Figure 2). The control was diapocynin (500 ng/mL) spiked in mouse plasma, which showed a clean single symmetrical peak appeared at a retention time of approximately 7 minutes. However, diapocynin was not detected in our samples of plasma, brain, and liver homogenates after iv bolus injection of apocynin. Our data showed clearly that diapocynin is not a metabolite of apocynin in vivo.

### 3.2 Time‐dependent distribution of apocynin in tissues and PK simulation

**FIGURE 3:**
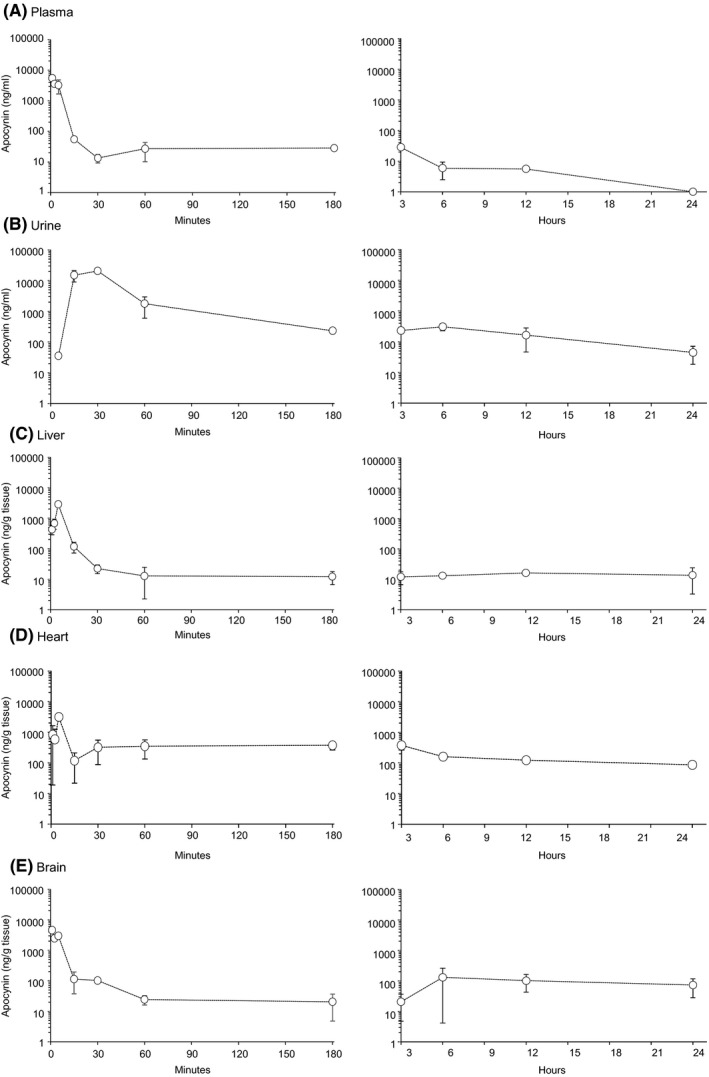
Pharmacokinetic profile of apocynin post intravenous bolus (5 mg/kg) in different tissues. (A) Plasma, (B) Urine, (C) liver, (D) Heart, and (E) Brain. Left panels showed PK profiles from time 1‐180 min; Right panels showed PK profiles from 3‐24 h. Data were presented as Mean ± SD. n = 3 mice per time point

Time‐dependent (up to 24h) tissue distributions of apocynin after iv injection were depicted in Figure 3. Apocynin was detected in the plasma with a concentration of 5494 ± 400 ng/mL at 1 minute, 3237 ± 1560 ng/mL at 5 minutes, ~50 ng/mL at 15 minutes and then fallen to ~10 ng/mL at 30 minutes after iv injection (Figure 3A). Apocynin was undetectable in the urine until 5 minutes after injection. Urine apocynin concentration reached peak (20492 ng/mL) at 30 minutes, and reduced to 230 ± 31 ng/mL at 3 hours after injection and remained detectable up to 12 hours (Figure 3B). Apocynin was detected in the brain tissues with a concentration of 4603 ± 208 ng/g of tissue weight at 1 minute and reduced to 103 ± 4 ng/g at 30 minutes after iv injection (Figure 3C). Apocynin was detected in the liver with a peak concentration of 2853 ± 35 ng/g tissue weight at 5 minutes after injection (Figure 3D). Apocynin was detected in the heart with a peak concentration of 3161 ± 309 ng/g tissue weight at 5 minutes after injection (Figure 3E).

The PK values of apocynin (1‐30 minutes) fits well with the linear regression model of log(time)‐concentration data (*R* ^2^ ≥.93) and were simulated using the Phoenix WinNonlin 8.1 non‐compartmental analysis model (Table 1). The central volume of distribution (*V*_d_) was 568.74 mL/kg. The *C*_max_ was 5494 ng/mL. Apocynin displayed rapid elimination (*t*_1/2_ = 0.05 hours) and systemic clearance (CL = 7.76 L/h/kg). Data collected at sampling time (1‐360 minutes) were used to fit into compartmental models in WinNonlin (Table 1). The smaller AIC and BIC values of the compartmental analysis indicate a better fit for the concentration‐time profile. However, when the sample number is small (n < 40), the smallest AICc should be used to predict the best fit for the model.^24^ Apocynin PK profile fitted better into the one compartmental model with the smallest values of AIC, AICc and BIC, 378.5, 379.7, and 382.4, respectively, in comparison to the values obtained for two and three compartment models.

**TABLE 1:** Pharmacokinetic parameters of apocynin simulated using WinNonlin 8.1 software (n = 3)

Pharmacokinetic parameters	Units	NCA	Time point: 0‐6 h		
			1‐CA	2‐CA	3‐CA	
*C*_0_	ng/mL	7359.89	—	—	—	
*C*_max_	ng/mL	5494.00	—	—	—	
AUC_0‐30min_	ng/mL/h	643.47	—	—	—	
AUC_0‐∞_	ng/mL/h	644.27	681.33	711.10	711.07	
*t*_1/2_	h	0.05	—	—	—	
Kel	1/h	13.65	—	—	—	
CL	mL/h/kg	7760.69	—	—	—	
tvCL1	mL/h	—	5541.14	5540.91	5521.18	
tvCL2	mL/h	—	—	0.17	4.03	
tvCL3	mL/h	—	—	—	16.45	
*V*_d_	mL/kg	568.74	—	—	—	
tvV1	mL	—	530.72	530.72	530.71	
tvV2	mL	—	—	0.37	63.99	
tvV3	mL	—	—	—	51.90	
LogLik	—	—	−186.25	−186.25	−186.25	
−2LL	—	—	372.50	372.50	372.50	
AIC	—	—	378.50	382.50	386.50	
AIC_c_	—	—	379.70	385.84	393.50	
BIC	—	—	382.04	388.39	394.75	
nParm	—	—	3	5	7	
nObs	—	—	24	24	24	

Abbreviations: 1‐CA, 2‐CA or 3‐CA, one, two or three compartmental analysis (values were from 0 to 6 h); AIC, Akaike information criterion; AICc, AIC corrected; AUC_0‐30 min_, Area under the curve from time 0 to 30 min; AUC_0‐∞_, Area under the curve from time 0 extrapolated to infinite time; BIC, Bayesian information criterion; *C*_0_, extrapolated plasma concentration at time 0; CL, clearance; *C*_max_, maximum plasma concentration; Kel, elimination rate constant; LogLik, log likelihood; NCA, non‐compartmental analysis (values were from 0 to 30 min, *R* ^2^ = .93); nObs, number of observations; nPharm, number of PK parameters; *t*_1/2_, terminal half‐life; tvCL1, tvCL2 or tvCL3, typical value (tv) of one, two and three compartmental clearance; tvV1, tvV2 or tvV3, typical value (tv) of one, two or three compartmental volume of distribution; *V*_d_, volume of distribution.

### 3.3 Pharmacodynamic investigation of apocynin on reducing organ ROS production

**FIGURE 4:**
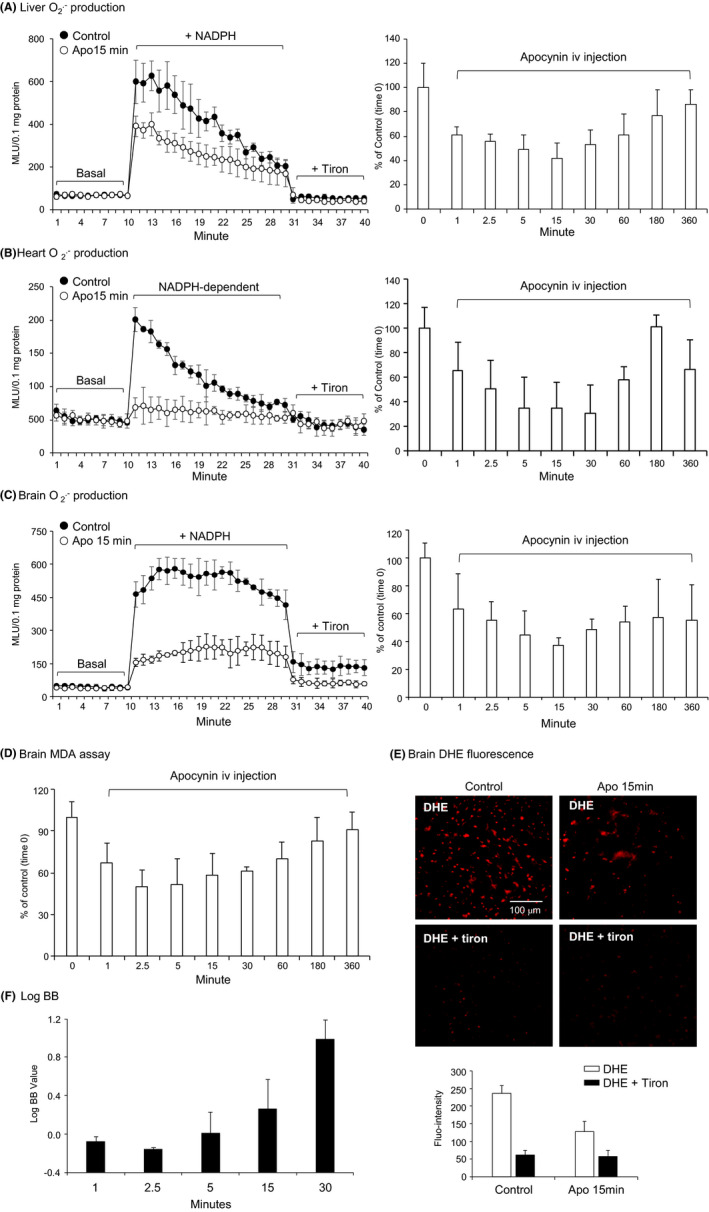
Pharmacodynamic profile of apocynin on inhibiting the levels of reactive oxygen species (ROS) production by major organs. (A) liver, (B) heart, and (C‐F) brain. A‐C, $O_{2}^{\cdot ‐}$ production in tissue homogenates measured by lucigenin‐chemiluminescence. Left panels: Representative kinetic detection of $O_{2}^{\cdot ‐}$ production in samples collected at 15 min after apocynin intravenous (iv) injection. Controls were tissue samples without apocynin injection. Right panels: Time course (0‐360 min). n = 3 mice per time point. D, Levels of lipid peroxidation in brain tissues detected by malondialdehyde (MDA) assay. E, In situ ROS production detected by dihydroethidium (DHE) fluorescence on brain sections at 15 min after iv injection of apocynin. Control samples were brain section of mice without apocynin. Tiron was used to confirm the detection of $O_{2}^{\cdot ‐}$. F, LogBB of apocynin after iv injection. Data were presented as Mean ± SD. n = 3 mice per time point

The time‐dependent effects of apocynin on the levels of organ $O_{2}^{\cdot ‐}$ production was examined by lucigenin‐chemiluminescence (Figure 4). The representative examples of real‐time recordings of $O_{2}^{\cdot ‐}$ production in samples collected at 15 minutes after iv injection were shown on the left panels, and the PD profiles of apocynin effects on tissue $O_{2}^{\cdot ‐}$ production were shown on the right panels. Tiron (an $O_{2}^{\cdot ‐}$ scavenger) was used to confirm the assay specificity.

In vehicle injected control mice, the levels of tissue $O_{2}^{\cdot ‐}$ production remained the same overtime (Figure S2). Compared to control samples (without apocynin, at time 0), apocynin inhibited the liver $O_{2}^{\cdot ‐}$ production within 1 minute, lasted for ~60 minutes and the effect disappeared at 3 hours after iv injection (Figure 4A, right panel). Apocynin inhibited the levels of heart $O_{2}^{\cdot ‐}$ production starting at 1 minute, becoming significant at 2.5 minutes, lasting for ~60 minutes and the inhibitory effect disappeared at 3 hours after iv injection (Figure 4B, right panel). Apocynin inhibited brain O_2_^.‐^ production (60.4 ± 25.4%) starting at 1 minute, becoming significant at 2.5 minutes, lasting up to 60 minutes and the inhibitory effect disappeared at 3 hours after iv injection (Figure 4C, right panel). Although the PK distributions were similar in these tissues, the PD profile of apocynin were different, which was due to the difference in Nox2 expression and activities. In the heart homogenates, most proteins were from cardiomyocytes where Nox2 expression/mg protein is relatively low. However, Nox2 is highly expressed in microglial cells, in brain microvascular system, and in neurons.

As an independent approach, the time‐dependent inhibitory effect of apocynin on brain ROS production was further examined by the levels of lipid peroxidation using an MDA assay (Figure 4D), which showed a similar pattern of inhibition by apocynin. We also examined the in situ ROS production using DHE fluorescence on brain sections harvested at 15 minutes after iv injection (Figure 4E). Apocynin inhibited effectively the DHE fluorescence on brain sections.

LogBB is defined as a reliable index of drug permeability of the BBB to reach the central nerve system (CNS).^22^, ^25^ If Log BB value is ≥0.3, it indicates the drug has readily crossed BBB and entered the CNS; and if the value is <−1, it suggests that the drug is poorly distributed to the brain.^25^ Apocynin had a LogBB value between −0.1 (at 1 minute) and 1 (at 30 minutes) (Figure 4F), which indicated that apocynin could easily cross the BBB and enter the CNS.

### 3.4 Effect of apocynin on reducing organ oxidative stress‐associated with metabolic disorders and aging

**FIGURE 5:**
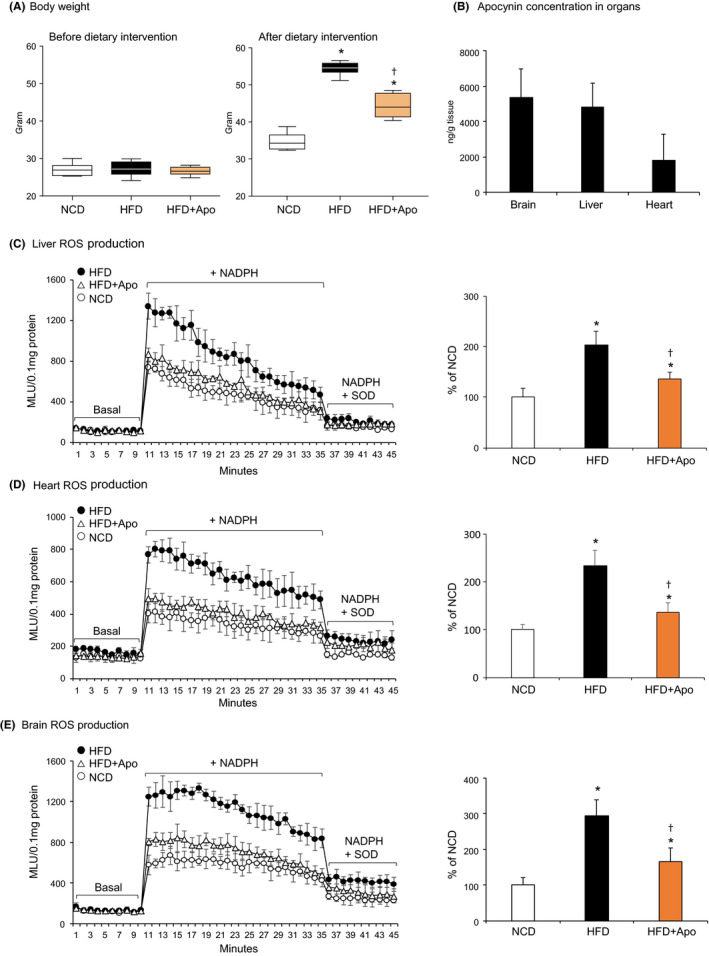
Effect of apocynin on reducing major organ (liver, heart, and brain) oxidative stress associated with dietary obesity and accelerated aging in mice. Apocynin was supplied in drinking water during high‐fat diet (HFD) period. A, Changes in bodyweight. B, Apocynin detected in tissue homogenates by HPLC‐MS/MS after treatment. C‐E, The levels of $O_{2}^{\cdot ‐}$ production in tissue homogenates detected by lucigenin‐chemiluminescence. Left panels: Representative kinetic detection of $O_{2}^{\cdot ‐}$ production. Right panels: Statistical analysis. Data were presented as % of NCD (100%). n = 6 mice/group. *P < .05 for indicated values vs NCD values. †*P* < .05 for indicated values vs HFD values. HPLC‐MS/MS, HPLC coupled to a quadrupole‐linear ion‐trap tandem mass spectrometry; NCD, normal chow diet

The therapeutic potential of apocynin on reducing major organ oxidative stress associated with metabolic disorders and aging was examined using a mouse model of HFD‐induced obesity and accelerated aging (Figure 5). Apocynin was supplied in drinking water (5 mmol/L) throughout the 16 weeks of HFD period. Mice were sacrificed at 11 months of age. Compared to controls, HFD mice (without apocynin treatment) had a massive increase in body weight and this was significantly reduced in the apocynin treatment group (Figure 5A). Apocynin was detected by HPLC‐MS/MS in major organs, that is, brain (5369 ± 1612 ng/g), liver (4818 ± 1340 ng/g), and heart (1795 ± 1487 ng/g) at the end of treatment (Figure 5B). These data further demonstrated that apocynin (oral application) penetrated easily through the BBB with a concentration in the brain similar to the concentration detected in the liver.

**FIGURE 6:**
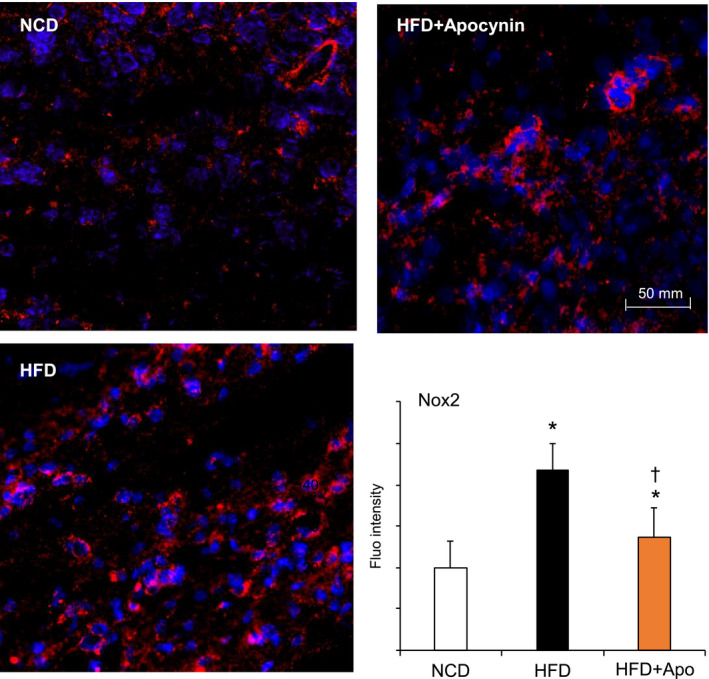
Brain Nox2 expression detected by immunofluorescence. Nox2 was labelled by Cy3 (red) and nuclei were labeled with 4′,6‐diamidino‐2‐phenylindole4′,6‐diamidino‐2‐phenylindole (DAPI, blue) to visualized cells. Nox2 fluorescence intensities were quantified. *P < .05 for indicated values vs NCD values. †*P* < .05 for indicated values vs HFD values. n = 6 mice/group and at least three sections per mouse brain were used. Nox2, Nox2‐containing NADPH oxidase

The effect of apocynin on reducing HFD‐induced major organ oxidative stress was examined using lucigenin‐chemiluminescence (Figure 5C‐E). SOD was used to confirm the assay specificity. Representative real‐time recordings of $O_{2}^{\cdot ‐}$ production detected in livers, hearts, and brains were shown in the left panels, and the statistical analysis (n = 6 mice) were given in the right panels of Figure 5C‐E. Compared to the levels of $O_{2}^{\cdot ‐}$ detected in control samples (expressed as 100%), HFD increased significantly the levels of $O_{2}^{\cdot ‐}$ production in the livers (Figure 5C), hearts (Figure 5D) and brains (Figure 5E). The effect of apocynin treatment on brain Nox2 expression was examined by immunofluorescence on brain sections (Figure 6). Compared to control samples, the level of Nox2 expression was increased markedly in the HFD brain sections and this was significantly reduced after apocynin treatment.

## 4 DISCUSSION

Apocynin has emerged as a promising inhibitor of Nox2 NADPH oxidase used for animal models of human oxidative‐stress related diseases.^8^, ^9^, ^11^, ^26^ However, there were discrepancies in the literature regarding the way that apocynin reduces ROS production in cells and in tissues, and no study had investigated its PKPD profiles in major tissues. The current study by investigating apocynin PKPD in mice demonstrated, for the first time, that apocynin after iv injection was distributed quickly into major organs, that is, heart, liver, and the brain and inhibited ROS production in these organs. We provided the evidence that apocynin can readily cross BBB with logBB values between −0.04 and 1 and inhibited effectively brain ROS production. More importantly, we demonstrated, using a mouse model of HFD‐induced obesity and accelerated aging, the therapeutic potential of apocynin in reducing major organ oxidative stress under these disease conditions. Diapocynin was not detected in any of our samples after iv injection, which supported the notion that diapocynin is not a metabolite of apocynin in vivo.^17^, ^27^

Linear ion‐trap tandem mass spectrometer methodology had been employed for the quantification of apocynin in plasma and tissue samples.^17^ However, the retention time of apocynin detected in this previous report was extremely short (0.45‐0.50 minutes), leaving people questioning if the analyte used in that study had been retained and separated enough to achieve a convincing result. In addition, the starting time point of sampling was 5 minutes after iv injection, which missed the crucial time points for accurate PK assessment of a small cell membrane permeable chemical, like apocynin.^17^ Compared to this previous report, the HPLC‐MS/MS method used in the current study has the following advantages. (a) a later retention time that demonstrated actual interaction with the stationary phase; (b) a neat and sharp chromatographic peak for quantification; (c) a reasonably high level of sensitivity to detect apocynin to a concentration as low as 1 ng/mL. We applied sampling time point of 1, 2.5 and 5 minutes, which covered the short half‐life of apocynin (*t*_1/2_ = 0.05 hours) following iv administration and provided accurate PKPD profile of apocynin.

The distribution of the compounds between blood and brain is a very important criterion for potential drug candidate to exert desired therapeutic effect in the CNS, or in contrary, not to cross BBB to avoid undesirable side effects. Previous study using in vitro BBB penetrating assay had shown that apocynin had a BBB permeability coefficient of 4.95 ± 0.39 cm/s, which was better than temozolomide.^28^ In the current study, we found that apocynin displayed a satisfactory permeability of the BBB with logBB values between −0.04 to 1. In addition, we also found that apocynin appeared to have a longer half‐life in the brain than in the plasma. Further detailed investigation is needed to address the mechanism of apocynin to cross BBB and the involvement of uptake/ efflux transporter in the brain tissues. However, using three separate complementary techniques of ROS detection, that is, lucigenin‐chemiluminescence, lipid peroxidation, and DHE fluorescence on brain sections, we have demonstrated clearly a time‐dependent inhibitory effect of apocynin on brain ROS production.

Mouse is one of the mostly used species for experimental studies of human diseases. Our study had established an effective method for apocynin extraction and detection in mouse tissues. The dosing regimen and sampling times used in our study were appropriate for apocynin PKPD characterization. Apocynin is a small molecule with pKa value of 8.17 and log *P* value (partitioning coefficient in n‐octanol/water) of .83, which give apocynin easy access to the cell membrane and its target sites.^7^ We found that apocynin after iv injection displayed a short plasma terminal half‐life and high clearance and kel, indicating that apocynin can be removed from blood at a high rate and distributed into peripheral organs.

It was reported previously that 80% of the apocynin administered by intraperitoneal injection in rats was recovered in unchanged form in urine samples collected at 20 hours after injection.^26^ Apocynin treatment has been found to raise the renal blood flow, glomerular filtration rate, increases creatine clearance and to protect kidney from cyclosporine A induced nephrotoxicity.^29^ In the current study, we found that apocynin has a high renal clearance, which is an advantage of apocynin to inhibit renal oxidative stress. However, in the case of renal failure and dysfunction, apocynin dose needs to be carefully adjusted. In agreement with a previous study,^17^ we found that apocynin PK values fitted best into one compartment model. The crucial information of apocynin PKPD profile reported here can be used for prediction of apocynin plasma concentrations under different experimental conditions and help to design dosing regimen in clinical medicine. However, interspecies differences in drug metabolism and BBB should be considered for the application of our data.

HFD‐induced mouse model of obesity and insulin resistance has been shown to display an accelerated aging phonotype and increased levels of oxidative stress/damage in organ function.^30^ Therefore, it would be a good model for us to examine the therapeutic potential of apocynin to inhibit organ oxidative stress associated with metabolic disorder and aging. Once again, we discovered that long‐term oral application of apocynin (16 weeks in drinking water) reduced significantly HFD‐induced major organ oxidative stress.

In conclusion, our study had characterised the PKPD profiles of apocynin as a Nox2 inhibitor both in vivo and in silico. Apocynin displayed a shot half‐life, a rapid clearance from blood and effective distribution to major organs including the liver, heart, and brain. Apocynin inhibited the ROS production in major organs under HFD‐induced diseased conditions. The crucial information provided by the current study helps further development of apocynin as potential drug for the treatment of oxidative‐stress related diseases.

## CONFLICT OF INTEREST

None declared.

## AUTHOR CONTRIBUTION

F.L. contributed to data generation and analysis. L.M.F. contributed to manuscript preparation and data presentation. N.M. contributed to MS/MS data gathering. J.M.L. contributed to study supervision and manuscript finalization.

## Supporting information

Fig S1‐S2

## Data Availability

The data that support the findings of this study are available from the corresponding author upon reasonable request.
