# Supplementary material for: In vivo and in silico characterization of apocynin in reducing organ oxidative stress: A pharmacokinetic and pharmacodynamic study
Source: Pharmacol Res Perspect. 2020 Aug 5;8(4):e00635. doi: 10.1002/prp2.635 (PMC7406636; doi:10.1002/prp2.635)
Supplement: Supplementary file 1 — Fig S1‐S2 [file PRP2-8-e00635-s001.pptx]

## Slide 1
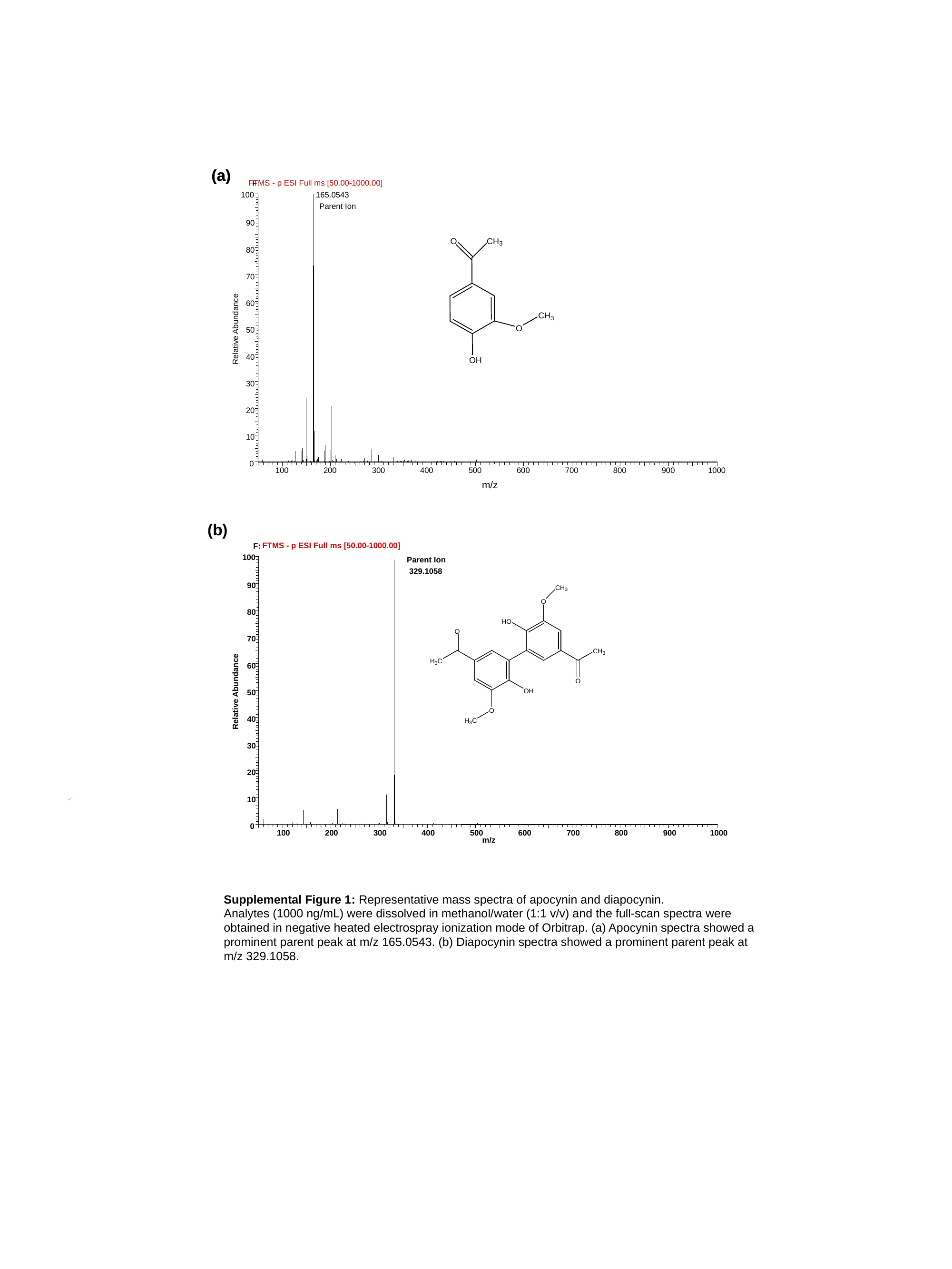

(a)
(a)
FTMS - p ESI Full ms [50.00-1000.00]
F:
165.0543
100
Parent Ion
90
80
70
60
50
Relative Abundance
40
30
20
10
0
100
200
300
400
500
600
700
800
900
1000
m/z
(b)
FTMS - p ESI Full ms [50.00-1000.00]
F:
100
Parent Ion
329.1058
90
80
70
60
Relative Abundance
50
40
30
20
10
0
100
200
300
400
500
600
700
800
900
1000
m/z
Supplemental Figure 1: Representative mass spectra of apocynin and diapocynin.
Analytes (1000 ng/mL) were dissolved in methanol/water (1:1 v/v) and the full-scan spectra were obtained in negative heated electrospray ionization mode of Orbitrap. (a) Apocynin spectra showed a prominent parent peak at m/z 165.0543. (b) Diapocynin spectra showed a prominent parent peak at m/z 329.1058.

## Slide 2
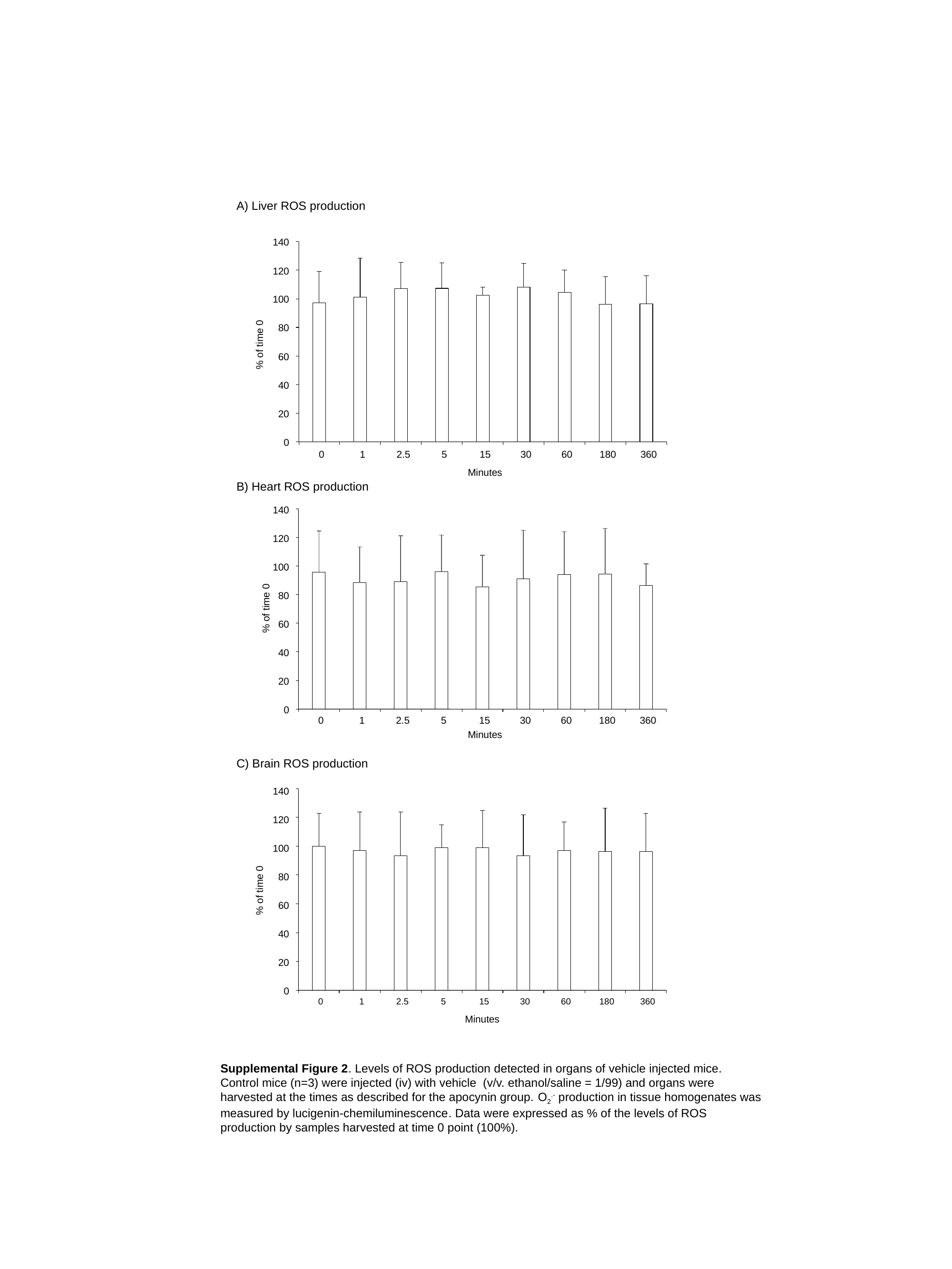

A) Liver ROS production
140
120
100
80
60
40
20
0
% of time 0
0
1
2.5
5
15
30
60
180
360
Minutes
B) Heart ROS production
140
120
100
80
60
40
20
0
% of time 0
0
1
2.5
5
15
30
60
180
360
Minutes
C) Brain ROS production
140
120
100
80
60
40
20
0
% of time 0
0
1
2.5
5
15
30
60
180
360
Minutes
Supplemental Figure 2. Levels of ROS production detected in organs of vehicle injected mice.
Control mice (n=3) were injected (iv) with vehicle (v/v. ethanol/saline = 1/99) and organs were harvested at the times as described for the apocynin group. O2.- production in tissue homogenates was measured by lucigenin-chemiluminescence. Data were expressed as % of the levels of ROS production by samples harvested at time 0 point (100%).
